# An observational study of compliance with the Scandinavian guidelines for management of minimal, mild and moderate head injury

**DOI:** 10.1186/1757-7241-20-32

**Published:** 2012-04-17

**Authors:** Ben Heskestad, Knut Waterloo, Tor Ingebrigtsen, Bertil Romner, Marianne Efskind Harr, Eirik Helseth

**Affiliations:** 1 Department of Neurosurgery, Oslo University Hospital - Ullevål, P.O. Box 4950, Nydalen N-0424, Oslo, Norway; 2 Department of Neurology, University Hospital of North Norway, N-9038 Tromsø, Norway; 3 Department of Psychology, Faculty of Health Sciences, University of Tromsø, N-9037 Tromsø, Norway; 4 CEO's office, University Hospital of North Norway, N-9038 Tromsø, Norway; 5 Institute for Clinical Medicine, Faculty of Health Sciences, University of Tromsø, N-9037 Tromsø, Norway; 6 Department of Neurosurgery, Neuroscience Centre, Rigshospitalet, University of Copenhagen, DK-2100 Copenhagen, Denmark; 7 Faculty of Medicine, University of Oslo, P.O.Box 1018, Blindern N-0315, Oslo, Norway

**Keywords:** Head injury, Guidelines, Implementation, Compliance

## Abstract

**Background:**

The Scandinavian guidelines for management of minimal, mild and moderate head injuries were developed to provide safe and cost effective assessment of head injured patients. In a previous study conducted one year after publication and implementation of the guidelines (2003), we showed low compliance, involving over-triage with computed tomography (CT) and hospital admissions. The aim of the present study was to investigate guideline compliance after an educational intervention.

**Methods:**

We evaluated guideline compliance in the management of head injured patients referred to the University Hospital of Stavanger, Norway. The findings from the previous study in 2003 were communicated to the hospitals physicians, and a feed-back loop training program for guideline implementation was conducted. All patients managed during the months January through June in the years 2005, 2007 and 2009 were then identified with an electronic search in the hospitals patient administrative database, and the patient files were reviewed. Patients were classified according to the Head Injury Severity Scale, and the management was classified as compliant or not with the guideline.

**Results:**

The 1 180 patients were 759 (64%) males and 421 (36%) females with a mean age of 31.5 (range 0-97) years. Over all, 738 (63%) patients were managed in accordance with the guidelines and 442 (37%) were not. Compliance was not significantly different between minimal (56%) and mild (59%) injuries, while most moderate (93%) injuries were managed in accordance with the guidelines (p < 0.05). Noncompliance was caused by overtriage in 362 cases (30%) and undertriage in 80 (7%). Guideline compliance was 54% in 2005, 71% in 2007, and 64% in 2009.

**Conclusions:**

This study shows higher guideline compliance after an educational intervention involving feed-back on performance. A substantial number of patients are exposed to over-triage, involving unnecessary radiation from CT examinations, and unnecessary costs from hospital admissions.

## Background

Clinical practice guidelines are developed to improve the quality of care by translating the best available scientific evidence into specific recommendations. Recent literature reviews suggests that guideline implementation improve not only the processes of patient care, but also health outcomes [1,2].

Initial management of mild head injuries is focused on the patient's risk for developing intracranial expansive lesions and early detection of deterioration in patients who initially seemed to have mild head injuries [3]. Studies in Scandinavia and Canada show significant inter- and intra-hospital variation in routines for assessment of the patient's consciousness level and for the use of radiological examinations [4-6].

The Scandinavian Neurotrauma Committee (SNC) published guidelines for management of minimal, mild and moderate head injured patients in 2000 [7]. In parallel, a number of guidelines and clinical decision-rules, such as the Canadian CT head rule (CCHR), the New Orleans Criteria and the National Institute for Health and Clinical Excellence (NICE) guidelines, were developed [8-10].

The publication of the SNC guidelines was followed by a national implementation process in Norway. A questionnaire based survey directed to departments responsible for head injury management indicated widespread use of the guidelines [11]. Our single-hospital study in 2003 showed compliance with the guidelines in only 51% of the cases [12]. This result was systematically communicated to the hospitals emergency room physicians repeatedly during the period 2003 to 2008. The present study aimed to investigate whether this intervention improved guideline compliance over time.

## Materials and methods

### Study region and population

The University Hospital of Stavanger is located in the south-western part of Norway and serves a population of 320 000. The hospital has a small neurosurgical unit, but head injured patients are primarily served by the department of general surgery. The minimal and mild head injuries are almost exclusively assessed and managed by pre registration house officers or senior house officers. A computed tomography (CT) scanner is available on a 24-hour basis.

An electronic search in the hospitals patient administrative database identified 1 180 patients with minimal, mild or moderate head injury (ICD-10 codes S00 through S09 with subgroups) referred to the hospital during the months January through June in the years 2005, 2007 and 2009. Patients were classified according to the Head Injury Severity Scale [13]. Patients with severe head injury (GCS score 3-8) were not included. The patient files were reviewed retrospectively, and trauma date, sex, age, Glasgow Coma Scale (GCS) score, use of CT and hospital admission, and the presence of eventual additional risk factors according to the SNC guidelines were registered (anticoagulation, clinical signs of skull fracture, shunt-treated hydrocephalus, multiple injuries, and posttraumatic seizures).

### Scandinavian guidelines for management of mild head injuries

**Figure 1:**
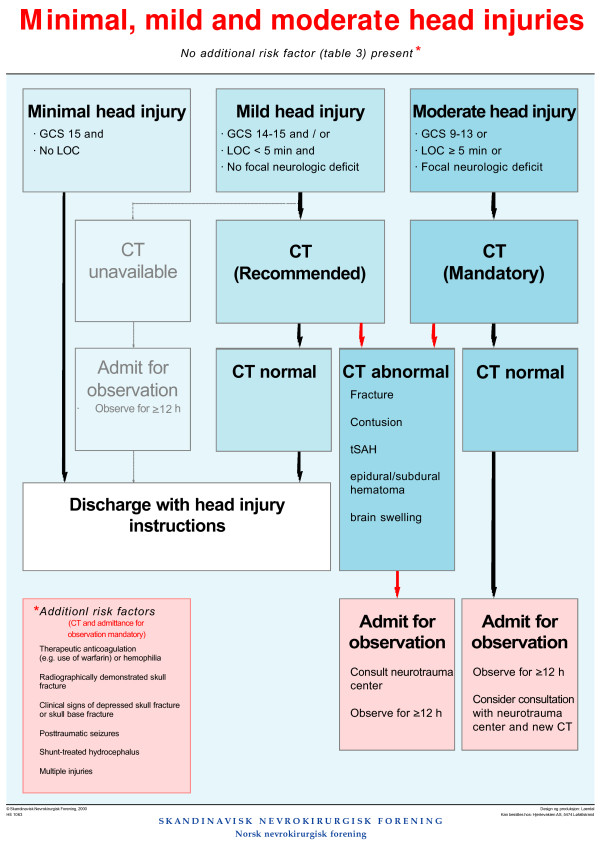
**Decision making algorithm for the management of minimal, mild and moderate head injuries recommended by the Scandinavian Neurotrauma Committee **[7].

The guideline was developed by the Scandinavian Neurotrauma Committee (SNC) and provides an evidence-based decision-making algorithm for management of minimal, mild and moderate head injuries (Figure 1.) [7]. For minimal head injuries, the guidelines recommend return to home without CT examination, unless additional risk factors are present. Patients with mild head injuries should undergo CT and return to home if the examination is normal, unless additional risk factors are present. For moderate injuries, CT and hospital admission should be provided.

### Implementation of the guidelines

The implementation of the guidelines in Norway included a secondary publication in the Journal of The Norwegian Medical Association which is distributed to 94% of the doctors in Norway. The guidelines were presented at the annual meeting of the Norwegian Society for Surgery in 2001 and the Trauma Care 2002 Congress in Stavanger. The guidelines are also regularly presented at annual mandatory courses in neurotrauma management for trainees in general and orthopedic surgery and during teaching of medical students in the four medical schools in Norway since 2001.

At Stavanger University Hospital, the guidelines were administratively implemented in 2001. The implementation process at the hospital included repeated lectures held every six months by consultant neurosurgeons and inclusion of the guidelines in the hospitals trauma manual.

### Intervention

Our study of physicians' guideline compliance from 2003 demonstrated a relatively low compliance with only 51% [12]. A substantial over triage with unnecessary CT examinations and hospital admissions generated unnecessary and inappropriate increases in cost of care, not poor patient outcomes. The publication, implementation and local announcement of the results is considered as an intervention in terms of a feed-back loop mechanism regarding improvement of physicians' guideline compliance.

### Classification of guideline compliance

The management of each single patient was classified as compliant with the guideline or not. Classification as compliant required correct use of CT and/or hospital admission in accordance with the guideline. The management was classified as non-compliant in cases of over-triage (unnecessary use of CT and/or admission) or under-triage (recommended CT examination and/or admission not performed).

### Statistics

We used SPSS for Windows (release 18.0; SPSS Inc., Chicago, IL) for statistical analyses. Comparisons of proportions were done with the chi-squared test. P-values < 0.05 were considered statistically significant.

## Results

The 1 180 patients were 759 (64%) males and 421 (36%) females with a mean age of 31.5 (range 0-99) years. The HISS classified 217(18%) patients with minimal, 806 (68%) with mild and 157 (13%) patients with moderate injuries.

**Figure 2:**
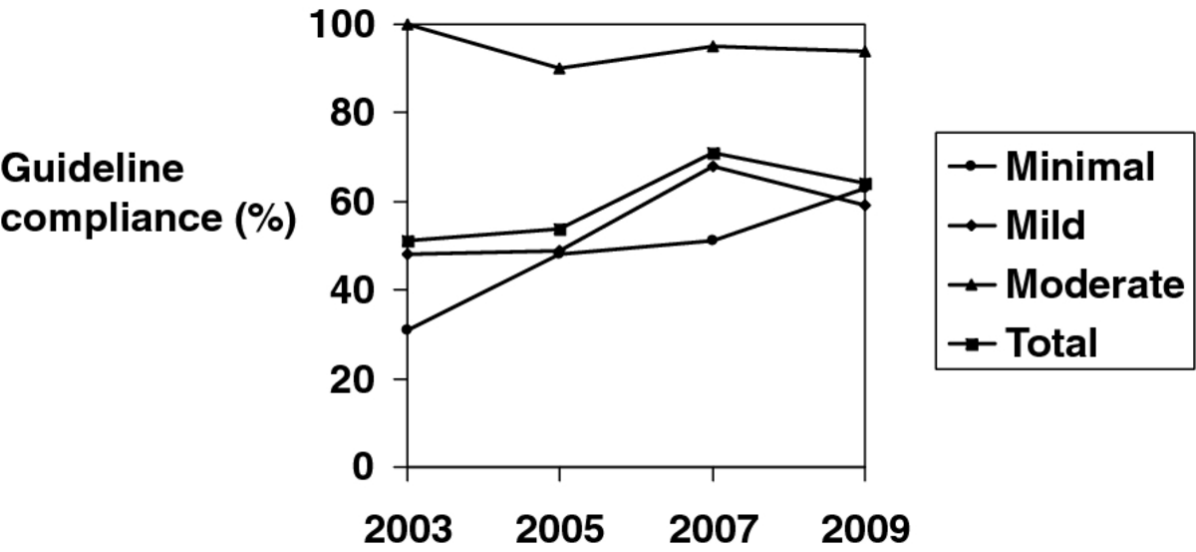
**The figure shows compliance with the Scandinavian guidelines for minimal, mild and moderate head injuries in the study years 2005, 2007 and 2009**. Observations from a previous study conducted in 2003 are included as a reference [12].

Table 1 show that 738 (63%) patients were managed in accordance with the guidelines, while 442 (37%) were not. Overall guideline compliance was 54% in 2005, 71% in 2007, and 64% in 2009 (Figure 2). The compliance was not significantly different between minimal (56%) and mild (59%) injuries, while most moderate (93%) injuries were managed in accordance with the guidelines (p < 0.05).

**Table 1:** Compliance with the Scandinavian Guidelines for Minimal, Mild and Moderate Head Injuries in 2005, 2007 and 2009

Head Injury Severity Scale classification	2005			2007			2009			Total		
	
	Compliant	Non-compliant		Compliant	Non-compliant		Compliant	Non-compliant		Compliant	Non-compliant	
	(n = 229)	(n = 192)		(n = 254)	(n = 104)		(n = 255)	(n = 145)		(n = 738)	(n = 442)	
								
		Overtriage	Undertriage		Overtriage	Undertriage		Overtriage	Undertriag		Overtriage	Undertriage
		(n = 167)	(n = 25)		(n = 86)	(n = 18)		(n = 108)	(n = 37)		(n = 362)	(n = 80)
Minimal HI (n = 217)	45 (48%)	46 (50%)	1 (1%)	29 (57%)	21 (41%)	1 (2%)	46 (63%)	27 (36%)	1 (1%)	120 (56%)	94 (43%)	3 (1%)
Mild HI (n = 806)	137 (49%)	122 (44%)	19 (7%)	171 (68%)	65 (26%)	14 (6%)	164 (59%)	81 (29%)	33 (12%)	472 (59%)	268 (33%)	66 (8%)
Moderate HI (n = 157)	47 (90%)	0 (0%)	5 (10%)	54 (95%)	0 (0%)	3 (5%)	45 (94%)	0 (0%)	3 (6%)	146 (93%)	0 (0%)	11 (7%)

Total (n = 1180)	229 (54%)	167 (39%)	25 (5%)	254 (71%)	86 (24%)	18 (5%)	255 (64%)	108 (27%)	37 (9%)	738 (63%)	362 (30%)	80 (7%)

HI; head injury

Noncompliance was caused by overtriage in 362 cases (30%) and undertriage in 80 (7%). In the minimal and moderate groups, noncompliance was almost exclusively caused by over- and under triage, respectively. Among patients classified with mild injuries, most (33%) non-compliant management was caused by over triage, but under triage also occurred in a substantial number of patients (8%). High guideline compliance in 2007 and 2009 was associated with lower over triage among patients classified with mild injuries (Table 1). The over triage caused 108 unnecessary CT examinations and 271 unnecessary hospital admissions among the 1 080 patients. Sixty nine patients did not undergo recommended CT and/or hospital admission.

## Discussion

This study was done after an educational intervention in 2004 aiming to improve compliance with The Scandinavian guidelines for management of minimal, mild and moderate head injuries in a Norwegian hospital. We observed an overall guideline compliance of 54% in 2005 and 64% in 2009. This is higher than the 51% reported in a previous study from the same hospital in 2003 [12]. The difference was caused mainly by lower over triage in the present study. It is disappointing that a substantial proportion (36%) of the patients continue to receive management not in compliance with guidelines. They are exposed to radiation from unnecessary CT-examinations, and the hospital is overspending sparse resources.

The major strengths of the present study were registration of guideline compliance at multiple time points, and at the level of each single patient, in one institution. This ensures reliable information on how patients were managed. The method does not allow generalization to other hospitals. Taking the major implementation efforts at Stavanger University Hospital into consideration, we find it unlikely that compliance rates would be higher at other hospitals. It is a weakness that the study design did not identify the underlying time trend. A statistical interrupted time series analysis could therefore not be done [14]. Clear conclusions on possible causal relations between the educational interventions and the time trend can therefore not be made. Further, it would have been of interest to analyse whether guideline compliance influenced patients' outcomes, especially with regard to intracranial complications. We do not report such data because the number of patients included is insufficient for such analysis.

The most accepted guidelines for management of minor head injuries include the Canadian CT head rule, the New Orleans criteria and the NICE-recommendations [8-10]. To our knowledge, only one study on compliance with these guidelines has been published [15]. This study demonstrated that after the implementation of NICE 2007 guidelines, a significant increase in compliance was observed for adult head injury patients. Few reports on head and brain injury guideline compliance have been reported. In a questionnaire based survey of compliance with the Brain Trauma Foundations guidelines for management of severe traumatic brain injury in the US, Hesdorffer and co-workers [16] reported a noncompliance rate of 35%. This represented an improvement from a non-compliance rate of 67% reported in a previous study from the same authors [17]. We suspect that the compliance rates would have been even lower if observations had been done on the level of single patients, as in the present study. Rusnak and co-workers [18] studied the use of the same guidelines in individual patients treated at five Austrian hospitals, and found that the proportion of patients being managed according to the different recommendations in the guidelines varied from 30 to 89%. Our studies show higher guideline compliance in 2009 than in 2003, but still 1 out of 3 patients are still not managed as recommended.

Advanced trauma life support (ATLS) principles are implemented at the hospital. These recommend a more extended use of CT and hospital admission in case of head injury. Interference between The Scandinavian guidelines and ATLS may be a significant contributor to overtriage. ATLS recommend hospitalization for head injured patients with GCS score 15 if there are significant other injuries or no companion at home. Our study shows that such patients are often also examined with CT. In our opinion, a reconsideration of the ATLS recommendations for head injured patients with GCS score 15 would be reasonable.

A recent study from the Nordic radiation protection co-operation reports concern about increased use of CT [19]. The use has increased gradually from about 50 examinations per 1 000 population per year in 1992 to between 60 (Finland), 82 (Denmark), 85 (Sweden), 145 (Iceland) and 195 (Norway) in 2008. The present study indicates that over-triage of head injured patients contribute to the increasing use of CT. Improved guideline implementation strategies and possibly re-evaluation of the applicability of the guidelines are therefore necessary.

Challenges with guideline implementation and adherence are well known problems across health care systems worldwide. A recent study by Flanagan and co-workers [20] differentiate between provider- and workflow-focused implementation strategies, and conclude that a combination of the two improve guideline compliance. We have used a provider focused approach, and possibly reached the highest possible result from this strategy. The findings from Flanagan and co-workers suggest that workflow-focused methods such as computer based reminder systems (tailoring) and redefined roles for physicians and other staff may be necessary to achieve further improvement. A more focused approach, based on analysis of barriers of adhering to individual recommendations may also improve the use and effectiveness of guidelines [21].

## Conclusions

This single center study shows that the compliance with the SNC guidelines for management of head injuries was 51% in 2003 and 64% in 2009, probably as a result from educational interventions involving feed-back on performance. A substantial number of patients are exposed to overtriage, involving unnecessary radiation from CT examinations, and unnecessary costs from hospital admissions. Future studies should focus on identification and analyses of barriers to guideline adherence.

## Competing interests

Two of the authors (T. I. and B. R.) were involved with the development of The Scandinavian guidelines for management of minimal, mild and moderate head injuries.

## Authors' contributions

BOH designed the study, acquired and analysed the data, and drafted the manuscript. KW participated in analysis and interpretation of the data and completion of the manuscript. TI contributed to the design of the study and participated in analysis and interpretation of the data and completion of the manuscript. BR participated in analysis and interpretation of the data and completion of the manuscript. MEH participated in acquisition of the data and drafting of the manuscript. EH participated in analysis and interpretation of the data and completion of the manuscript, and participated in designing the study. All authors read and approved the final manuscript.
